# 

**Published:** 1906-06-02

**Authors:** 


### BOOKS RECEIVED.

Mellins Food, Ltd.
"The Care of Infants."

W. B. Saunders and Co.
"Nursing in the Acute Infectious Diseases," by G. P. Paul, M.D.

Henry Kimpton.
"The Mother's Guide to the Feeding and Rearing of Children," 3rd edition, by T. Dutton, M.D.

J. B. Lippincott Co.
"International Clinics," Sixteenth Series, Vol. 1.

Baillière, Tindall, and Cox.
"Gastric Surgerv." By Herbert J. Paterson, M.A., M.B.
"New Serum Therapy." By D. Montgomerie Paton, L.R.C.S.

The F. A. Davis Company, Philadelphia.
"Nose and Throat Surgery." By Beaman Douglass, M.D.
"The Test Diet in Intestinal Diseases." By Professor Dr. A. Schmidt and C. D. Aaron, M.D.

G. Routledge and Sons, Limited.
"The Management of Children." By Howard Barrett, M.R.C.S.

The Scientific Press, Limited.
"The Consumptive Working Man." By Noel D. Bardswell, M.D.

J. Wright and Co., Bristol.
"Diabetes Mellitus." By Professor Dr. C. Van Noorden.

### Medical Appointments and Vacancies.

### VACANCIES.

Bethlem Hospital.—Two Resident House Physicians, unmarried.

Birmingham Workhouse Infirmary.—Assistant Resident Medical Officer.

Bristol Royal Hospital for Sick Children and Women.—House Surgeon.

City of London Hospital for Diseases of the Chest, Victoria Park, E.—House Physician. Also Surgeon Dentist.

County of London.—Woman Inspector under the Midwives Act, 1902.

Coventry and Warwickshire Hospital, Coventry.—Honorary Medical Officer.

Devon County Asylum.—Third Assistant Medical Officer.

East London Hospital for Children and Dispensary for Women, Shadwell, E.—Assistant Surgeon.

Leicester Infirmary.—Assistant House Surgeon.

London Hospital, Whitechapel, E.—Surgical Registrar.

Manchester, St. Mary's Hospital for Women and Children. Medical Officer.

North-Eastern Hospital for Children, Hackney Road, Bethnal Green, E.—Resident Medical Officer. Also Pathologist and Bacteriologist.

Northumberland House Private Asylum, Finsbury Park, N. Assistant Medical Officer.

Plymouth, South Devon and East Cornwall Hospital.—Assistant House Surgeon.

Rochford Union.—District Medical Officer, Public Vaccinator, and Workhouse Medical Officer.

Royal Hospital for Diseases of the Chest, City Road, E.C.—Medical Officer to the Röntgen Ray Department.

St. Marylebone General Dispensary, Welbeck Street, Cavendish Square.—Honorary Dental Surgeon.

Victoria Hospital for Children, Tite Street, Chelsea, S.W.—Surgeon to Out-patients.

West London Hospital, Hammersmith Road, W.—House Physician.

The Earl Cawdor as Treasurer of the London Homœopathic Hospital, Great Ormond Street, W.C., has received £250, being a donation in aid of its funds from the Treasurer of Smith's Charity. The hospital is appealing for £30,000 for extending the hospital on its own freehold ground adjoining, and Sir Henry Tyler, the Chairman of the House Committee, has promised £10,000, and Lord Dysart £2,000 to the appeal.

Great interest has been caused in professional circles at Newcastle-on-Tyne by the approaching visit of the King and Queen on July 11 to open the new wing of the Armstrong College and the Royal Victoria Infirmary.

The University of Durham has conferred the degree of D.C.L. *honoris causa* upon Baron Takaki, Surgeon-General of the Imperial Japanese Navy, who recently gave an address to the medical profession in the North of England in the University of Durham College of Medicine.

Mr. W. Rae, senior partner in one of the oldest and largest practices at Northampton, died last week. Dr. Rae was deservedly popular, his kindness to his patients and to all with whom he came in contact being proverbial. Dr. Rae was a bachelor, and he has left practically the whole of his estate to the charities of Northampton. The Victoria Nursing Institute will obtain the largest share, but the General Hospital, the Royal Victoria Dispensary, the Blind Association, and the Good Samaritan Society are also to benefit considerably by this bequest.

### NOTICE TO CORRESPONDENTS.

All MSS., Letters, Books for Review, and other matters intended for the Editor should be addressed The Editor, "The Hospital," The Hospital Buildings, 28 & 29 Southampton Street, Strand, W.C.

The Editor cannot undertake to return rejected MS., even when accompanied by stamped directed envelope.

**Notice to Advertisers and Subscribers.**

All Advertisements, Orders for Copies of The Hospital, and Business Communications should be addressed to **THE MANAGER (Not to the Editor),** The Hospital, 28 & 29 Southampton Street, Strand London, W.C.

The Cost of Subscription, post free, is as follows:—Per week, 3½d.; per quarter, 3s. 3d.; per half-year, 6s. 6d.; per annum, 13s. Foreign and Colonial:—Per annum, 19s. 6d., payable, in all cases, in advance.

**NOTICE.—Vol. XXXIX. of "The Hospital" (October 1905 to March 1906), in handsome cloth binding, will shortly be on sale at the office of this Journal, price 10s. 6d. Cases for binding the Numbers contained in this Volume 2s. Index to Vol. XXXIX., 3½d., post free.**

**The Monthly Part for June is now ready. Price 1s., by post, 1s. 3d.**

### Tenders.

THE

**CANCER HOSPITAL (FREE),**

**FULHAM ROAD, S.W.**

THE Committee of Management are prepared to receive TENDERS for the Supply of Meat, Bread, Groceries, Cheesemongery, Milk, Fish, Ale and Stout, Coal and Coke.

Forms of Tenders, which must be returned filled in on or before noon TUESDAY, June 12, can be obtained from the undersigned,

FRED. W. HOWELL, Secretary.

ONE SHILLING

### Shilling Nursing Handbooks

ONE SHILLING

POST FREE, 1/1.

**HOW TO BECOME A CERTIFIED MIDWIFE.**
By E. L. C. APPEL, M.B., B.S., B.Sc.Lond.

**THE NURSING OF SICK CHILDREN.**
By Dr. BURNET.

**SIMPLE INTRODUCTORY LESSONS IN MIDWIFERY.**
By M. LOANE.

**THE MODERN NURSING OF CONSUMPTION.**
By Dr. JANE H. WALKER.

**THE EXAMINATION OF THE URINE.**
By J. K. WATSON, M.D., M.B., C.M., Author of "A Handbook for Nurses."

**SIMPLE SANITATION:**
**The Practical Application of the Laws of Health to Small Dwellings.**
By M. LOANE. With an Introductory Chapter on Sanitary Legislation by Dr. A. MEARNS FRASER, Medical Officer of Health, Portsmouth.

**HINTS ON HOW TO START A DISTRICT NURSING ASSOCIATION.**
By A COUNTY SUPERINTENDENT.

**SOME HEALTH ASPECTS OF EDUCATION.**
By PERCY G. LEWIS, M.D., M.R.C.S., L.S.A., Author of "Nursing: Its Theory and Practice."

**THE COMMONWEALTH OF THE BODY.**
Papers for Young People.
By G. A. HAWKINS-AMBLER, F.R.C.S., Author of "Health Gossips for Women."

**THE NURSE IN HOT CLIMATES.**
By Dr. E. HENDERSON.

**PRACTICAL HINTS ON DISTRICT NURSING**
By Miss AMY HUGHES.

**HOSPITAL AND PRIVATE NURSING.**
By Miss S. E. ORME.

**THE MIDWIVES' POCKET BOOK.**
With Prefatory Chapter on the Midwives Bill.
By Miss HONNOR MORTEN, Author of "The Nurses' Dictionary," &c. &c.

**FEVERS AND INFECTIOUS DISEASES:**
**Their Nursing and Practical Management.**
By WILLIAM HARDING, M.D.Ed., M.R.C.P.Lond., Author of "Mental Nursing," &c.

**NOTES ON PHARMACY AND DISPENSING FOR NURSES.**
New and Revised Edition.
By C. J. S. THOMPSON.

**MENTAL NURSING.**
By WILLIAM HARDING, M.B.Ed., M. R.C.P.Lond.

**SICK NURSING AT HOME.**
By Miss L. G. MOBERLEY.

**GYNÆCOLOGICAL NURSING.**
By G. A. HAWKINS-AMBLER, F.R.C.S., Author of "The Commonwealth of the Body."

**SYLLABUS OF LECTURES TO NURSES.**
By ANDREW DAVIDSON, M.D.

**The Scientific Press, Ltd.,**

☞ *Catalogue of Nursing Manuals sent post free on application.* ☜

**Publishers of Nursing Text-Books, Charts, and Case-Books of all Descriptions.**

**28 & 29 SOUTHAMPTON STREET, STRAND, LONDON, W.C.**

### NORTH-EASTERN HOSPITAL FOR CHILDREN,

### "THE UNIFORM SYSTEM OF ACCOUNTS."

BY

### SIR HENRY BURDETT, K.C.B.

THE

### INDEX OF CLASSIFICATION,

Whereby every item of expenditure may be dealt with under identical heads by every group of Institution is now published separately.

**Price 1s.; 1s. 1d. post free.**

THE SCIENTIFIC PRESS, LTD.,
28 and 29 Southampton Street, Strand, London, W.C.

